# The impact on carers of individuals with social care needs: the Whole Systems Demonstrator study

**Published:** 2012-06-15

**Authors:** Michelle Beynon, Shashi Hirani, Martin Cartwright, Lorna Rixon, Helen Doll, Catherine Henderson, Stanton Newman

**Affiliations:** School of Health Sciences, City University, UK; School of Health Sciences, City University, UK; School of Health Sciences, City University, UK; School of Health Sciences, City University, UK; University of East Anglia, UK; LSE Health and Social Care, London School of Economics, UK; School of Health Sciences, City University, UK

**Keywords:** carers, telecare, quality of life, carer outcomes, social care needs

## Abstract

**Introduction:**

Carers of individuals with social care needs (including frail elderly people, those at risk of falls and those requiring night sitting), are subject to high levels of carer burden, social isolation and poor psychological outcomes. Carers’ worries and concerns include leaving the individual alone; risks of harm to the care-recipient; or household emergencies in their absence. At present there is a lack of research on the impact of telecare interventions on carer outcomes. It is feasible that carers’ anxieties and level of strain may be ameliorated with concomitant increases in quality of life, with the introduction of telecare systems for their care-recipient.

**Objectives:**

This study investigates the secondary impact of telecare on carers, specifically whether the use of telecare reduces informal carer burden and social isolation and whether it improves carers’ psychological well-being and confidence in leaving the care-recipient alone.

**Design:**

We report on the prospective analysis of a pragmatic controlled trial on carers of telecare users and controls within the WSD evaluation of telecare (a pragmatic cluster randomised controlled trial investigating the effectiveness of telecare for social care recipients).

**Methods:**

Carers of individuals with social care needs were identified and recruited to the study via a combination of snowball sampling, light touch visits (to users) and self selection. Over 200 carers completed a questionnaire pack at baseline, and at short and long-term follow-ups; with roughly equal numbers of carers of recipients of telecare and recipients of usual care. The questionnaire pack included measures of quality of life (ICECAP), health related quality of life (SF12, EQ5D), psychosocial well-being (STAI-6, CESD10) and care-giver strain measures (CGSI, Carer confidence and anxiety).

**Results and Conclusions:**

Multi-level modelling of difference in outcomes between carer groups and across time was conducted with appropriate covariates. Specific findings from the Telecare Carers Study are embargoed until these analyses have been peer-reviewed and accepted for publication.

